# Pathophysiological Role of Transient Receptor Potential Mucolipin Channel 1 in Calcium-Mediated Stress-Induced Neurodegenerative Diseases

**DOI:** 10.3389/fphys.2020.00251

**Published:** 2020-03-24

**Authors:** Giorgio Santoni, Federica Maggi, Consuelo Amantini, Oliviero Marinelli, Massimo Nabissi, Maria Beatrice Morelli

**Affiliations:** ^1^Immunopathology Laboratory, School of Pharmacy, University of Camerino, Camerino, Italy; ^2^Department of Molecular Medicine, Sapienza University, Rome, Italy; ^3^Immunopathology Laboratory, School of Biosciences and Veterinary Medicine, University of Camerino, Camerino, Italy

**Keywords:** neurodegenerative disease, TRPML1, lysosomal storage disease, oxidative stress, mitochondria, autophagy, Ca^2+^ signaling

## Abstract

Mucolipins (TRPML) are endosome/lysosome Ca^2+^ permeable channels belonging to the family of transient receptor potential channels. In mammals, there are three TRPML proteins, TRPML1, 2, and 3, encoded by *MCOLN1-3* genes. Among these channels, TRPML1 is a reactive oxygen species sensor localized on the lysosomal membrane that is able to control intracellular oxidative stress due to the activation of the autophagic process. Moreover, genetic or pharmacological inhibition of the TRPML1 channel stimulates oxidative stress signaling pathways. Experimental data suggest that elevated levels of reactive species play a role in several neurological disorders. There is a need to gain better understanding of the molecular mechanisms behind these neurodegenerative diseases, considering that the main sources of free radicals are mitochondria, that mitochondria/endoplasmic reticulum and lysosomes are coupled, and that growing evidence links neurodegenerative diseases to the gain or loss of function of proteins related to lysosome homeostasis. This review examines the significant roles played by the TRPML1 channel in the alterations of calcium signaling responsible for stress-mediated neurodegenerative disorders and its potential as a new therapeutic target for ameliorating neurodegeneration in our ever-aging population.

## Introduction

Neurodegenerative diseases entail progressive destruction and loss of neural cells and impairment of both motor and cognitive functions. They include Parkinson’s disease (PD), Alzheimer’s disease (AD) and Amyotrophic lateral sclerosis (ALS), as well as pathologies caused by lysosomal accumulation, such as Mucolipidosis type IV (MLIV) and Niemann-Pick disease (NPD).

All neurodegenerative disorders are marked by the accumulation of abnormally aggregated proteins and mitochondrial dysfunction. Some genes involved in PD or ALS are related to mitochondria, the main source of reactive oxygen species (ROS) in aging cells (Indo et al., 2015). In addition, aggregated misfolded proteins can inhibit mitochondrial functions and induce oxidative stress (Abramov et al., 2017).

Several studies demonstrated the importance of maintaining the balance between oxidative stress and the antioxidant system (Li et al., 2013). Under physiological conditions, low levels of ROS are required in processes such as inflammation, synaptic plasticity, learning and memory. On the other hand, high ROS levels are dangerous for the cells themselves, due to their high reactivity against biological structures (Kishida and Klann, 2007). In this regard, the central nervous system is particularly susceptible to oxidative stress and its related damage, because of high oxygen consumption and poor counteracting antioxidant defenses.

These antioxidant defenses are generally classified as enzymatic or non-enzymatic. Among the former are superoxide dismutase, catalase, glutathione peroxidase, and glutathione reductase, while the latter include glutathione, selenium and vitamins A, E and C (Rego and Oliveira, 2003).

Evidence indicates that transient receptor potential (TRP) channels play a central role in the modulation of oxidative stress and lysosome functions, in particular by regulating calcium ion influx and efflux (Sterea et al., 2018). In the TRP family, the mucolipin (TRPML) subfamily is of particular interest because it localizes to the endo-lysosomal compartment. The best-characterized member is TRPML1, encoded by the *MCOLN1* gene. TRPML1 is permeable to Ca^2+^, Na^+^, Fe^2+^, Mg^2+^, and K^+^ (Xu et al., 2007; Dong et al., 2008, 2009). It has an intraluminal loop that can be protonated activating the channel (Xu et al., 2007; Dong et al., 2008). It is activated by phosphatidylinositol-3,5-biphosphate (PtdIns(3,5)P2), voltage, low pH, and the synthetic compounds MK6-83 and ML-SA1 (Raychowdhury et al., 2004; Dong et al., 2010; Grimm et al., 2010; Shen et al., 2012; Zhang et al., 2012; Chen et al., 2014). It is inhibited by phosphatidylinositol-4,5-biphosphate (PtdIns(4,5)P2), sphingomyelins, and lysosomal adenosine (Shen et al., 2012; Zhang et al., 2012). Some studies indicate that TRPML1 is also involved in lysosomal storage, transportation and acidic homeostasis and in this way it promotes the cation efflux into the cytosol (Morgan et al., 2011). TRPML1 is also classified as an important regulator of autophagy, given that TRPML1 mutations affect lysosomal storage and lysosomal impairment is responsible for autophagy defects. TRPML1 can also be negatively regulated through the phosphorylation of Ser572 and Ser576 residues by the target of rapamycin (TOR) with a consequent autophagy decrease (Onyenwoke et al., 2015). Autophagy can target oxidized and damaged molecules for lysosomal degradation. ROS are able to induce autophagy and their major sources are mitochondria, localized in proximity of lysosomes (Elbaz-Alon et al., 2014; Li et al., 2015). Zhang et al. demonstrated that endogenous ROS are able to regulate lysosomal activities through the TRPML1 channel, which functions as a “ROS sensor” (Zhang et al., 2016). In this way, lysosomal Ca^2+^ release induces nuclear translocation of transcription factor EB (TFEB) (Medina et al., 2015), followed by autophagosome and lysosome biogenesis, induction of autophagic flux and re-establishment of redox homeostasis. Hence, we are interested in the interplay between TRPML1, calcium flux and neurodegenerative diseases.

There are two other members in the TRPML subfamily, TRPML2 and TRPML3, encoded by *MCOLN2* and *MCOLN3* genes. Like TRPML1, they are active in late endosomes/lysosomes; in addition, TRPML2 and TRPML3 are active in early endosomes, and TRPML2 also in recycling (Chen et al., 2017; Plesch et al., 2018). They have not been correlated with neurodegeneration in humans so far.

The aim of this review is to highlight the role of TRPML1 in neurodegenerative diseases, reporting the current data available in the literature. The following sections describe some of the most important neurodegenerative diseases, with attention to the role of TRPML1 functions.

## Alzheimer’s Disease

Alzheimer’s disease (AD) is a neurodegenerative disorder characterized by marked cognitive disabilities, ranging from memory loss to synapse disappearance. Pathologic changes occur in the brain such as pyramidal neuron damage, extracellular accumulation of β-amyloid aggregates and neurofibrillary tangles containing hyperphosphorylated Tau protein (Selkoe, 2001). A central hallmark of AD pathogenesis is Ca^2+^ dyshomeostasis. Mutations in the β-amyloid precursor protein (APP) or in presenilin (PS) 1/2, characteristics of familial AD, are associated with aberrant Ca^2+^ concentrations responsible for apoptosis and excitotoxicity in neurons (Yamamoto et al., 2007). In particular, models of AD show an atypical efflux of lysosomal Ca^2+^, which leads to impaired autophagy, a process in which lysosomes degrade proteins or cytoplasmic organelles (Komatsu et al., 2006). Autophagy also contributes to β-amyloid secretion and metabolism, and its dysfunction is associated with the induction of neuronal lesions (Nixon, 2017). Related to autophagy, anomalies of the endosomal-lysosomal network are characteristic of AD. Studies performed in PS1 mutated neurons demonstrated that the loss of PS1 disrupts lysosome acidification and thus impairs autophagy.

In APP/PS1 transgenic mice, neuronal TRPML1 is downregulated, the mTOR pathway is inhibited and beclin and LC3 protein upregulated. Conversely, TRPML1 overexpression triggers autophagy by activating the mTOR pathway (Zhang et al., 2017) thus diminishing neuronal apoptosis. When primary neurons, isolated from hippocampus of APP/PS1 transgenic mice, were treated with β-amyloid peptides, cell viability was impaired and lysosomal Ca^2+^ concentration was reduced. The upregulation of TRPML1 expression is able to strongly attenuate these effects, and thus it is possible that the channel plays an important role in the maintenance of lysosomal homeostasis (Zhang et al., 2017).

Lee and coworkers demonstrated that PS1 knock-out (KO) cells, used as model of early AD, display elevated lysosomal pH due to vATP-ase deficiency. This alkaline lysosomal pH inhibits the function of the two-pore channel 2 (TPC2) and stimulates an abnormal TRPML1-mediated depletion of lysosomal Ca^2+^ (Lee et al., 2015). Their results indicated that the endogenous TRPML1 is present in a hyperactive state in PS1 KO cells and contributes to Ca^2+^ efflux from lysosomes thus leading to autophagy impairment. In addition, the observation that treatment of PS1 KO cells with an inhibitor of NAADP-dependent channels resets Ca^2+^ homeostasis suggests that there is a complex interplay between TRPML and NAADP signaling. However, normalization of Ca^2+^ levels is not able to reverse proteolytic and autophagic defects in PS1 KO cells. Rather, the associated changes in lysosomal pH appear to be more functionally significant. No data are reported in this study about changes in β-amyloid peptide ratio, production and clearance; thus, the involvement, in β-amyloid alterations, of the lysosomal Ca^2+^ efflux evoked by TRPML1 has not been clarified so far in PS1 KO cells.

However, in a triple transgenic gp120/APP/PS1 mouse model, a role of TRPML1 in the regulation of β-amyloid peptide clearance has been suggested. In fact, there is evidence in the brain of HIV-infected patients that β-amyloid peptides accumulate causing cognitive deficits that overlap with those of the AD. It has been demonstrated that the viral protein gp120 promotes the accumulation of β-amyloid peptides, sphingomyelin and Ca^2+^ inside lysosomes and autophagic compartments. The activation of TRPML1, by its agonists, induces Ca^2+^ efflux from lysosomes with consequent pH acidification that promotes the clearance of intraneuronal β-amyloid/sphingomyelin deposits (Bae et al., 2014). So, these findings showed that the induction of lysosomal acidification by activating the TRPML1-induced Ca^2+^ efflux reduces the deposition of β-amyloid peptides in the HIV-infected brain.

Among the potential factors implicated in AD, an impairment of the blood brain barrier (BBB), responsible for the increase in LDL flux from the peripheral circulation into the brain, has been described. Moreover, high plasma levels of cholesterol are found to be able to compromise the BBB. Once inside the brain, LDL can enter into endolysosomes and deacidify them, thus blocking their function. This mechanism is responsible for the LDL-induced increases in β-amyloid peptides generation in neurons. It has been demonstrated that the TRPML1 agonist ML-SA1 is able to prevent LDL-induced increases in β-amyloid peptides, while TRPML1 silencing potentiates LDL-induced effects (Hui et al., 2019).

## Parkinson’s Disease

Parkinson’s disease (PD) is characterized by the progressive degeneration of the dopaminergic neurons located in the substantia nigra pars compacta (SNc) (Lima et al., 2009). The main hallmarks of PD are progressive neuronal loss and intracellular inclusions known as Lewy bodies and neurites, predominantly composed of misfolded and aggregated forms of α-synuclein (Lotharius and Brundin, 2002). The causes involved are mitochondrial dysfunction and oxidative stress supported by *PTEN-induced kinase 1*, *Parkin*, *Protein deglycase-1*, and *Leucine-rich repeat kinase 2 (LRRK2)* genes that regulate mitochondrial and ROS homeostasis (Kilpatrick, 2016).

Recent studies have reported that in Parkinson’s disease, the mitochondrial Ca^2+^ dynamics are altered when impaired formation of membrane connections between mitochondria and the endoplasmic reticulum (ER) or other components of Ca^2+^ signaling cause neurodegeneration in SNc neurons, which are already vulnerable due to excessive Ca^2+^ influx. Indeed, SNc neurons are subjected to an excessive influx of Ca^2+^ through voltage-gated calcium (Cav1.3) channels (Guzman et al., 2009). This Ca^2+^ exposure comes at an energetic cost to mitochondria. As a result, neurons experience oxidative stress, which might make them less tolerant to stressors (Guzman et al., 2010).

Since mitochondria, ER and lysosomes communicate through Ca^2+^ signals, and since TRPML1, like other endo-lysosomal Ca^2+^ channels, crosstalks with ER Ca^2+^ channels, it may be that alterations in TRPML1 activity contribute to PD (Kilpatrick, 2016).

Lysosomes are also involved in endocytic, autophagic and secretory pathways. Since lysosomes degrade α-synuclein through chaperone-mediated autophagy (CMA) (Cuervo et al., 2004), the accumulation of α-synuclein implicates lysosomal dysfunction in PD. Lysosomal Ca^2+^ content is impaired in a beta-glucocerebrosidase GBA1-mutated PD model and is related to altered endo-lysosomal morphology. In addition, the LRRK2-mutated PD model shows deregulated lysosomal Ca^2+^ signaling and altered morphology. It has been suggested that excessive activation of TRPML channels, caused by changes in lysosomal pH, depletes lysosomal Ca^2+^ (Lee et al., 2015). If this is the case, then the increased NAADP-evoked Ca^2+^ signals measured in LRRK2-mediated PD (Hockey et al., 2015) probably drain the lysosomes of Ca^2+^.

Moreover, in a PARK9-mutant PD model, the loss of lysosomal type 5 P-type ATPase function leads to α-synuclein accumulation. Indeed, PARK9 regulates lysosomal exocytosis, a pathway that could be potentiated to reduce α-synuclein accumulation. Tsunemi demonstrated that TRPML1 agonists are able to increase lysosomal exocytosis, thus impairing α-synuclein intracellular levels (Tsunemi et al., 2019).

Since neuroinflammation seems to be essential for PD pathogenesis (Whitton, 2007; Ransohoff, 2016), Gao et al. (2003) conceived a PD mouse model based on treatment with the inflammogen lipopolysaccharide (LPS) plus the neurotoxin 1-methyl-4- phenyl-1,2,3,6-tetrahydropyridine (MPTP). In this model, NADPH-oxidase-dependent ROS generation has a central role (Gao et al., 2003). Through metabolomics analysis, Huang et al. exploited the LPS-MPTP model to identify adenosine and adenosine deaminase (ADA) as the most promising therapeutic targets for PD (Huang et al., 2019). Previously it had already been demonstrated that the neuromodulator adenosine is able to weaken oxidative stress and excitotoxicity (Fredholm, 2007; Min et al., 2008). However, its use is limited by several adverse side effects, rapid metabolism and difficulty in penetrating the blood brain barrier (Phillis and Wu, 1981). Therefore, increasing its local release through the inhibition of ADA could be a promising approach. Indeed, compared to controls, mice exposed to MPTP have impaired adenosine concentration and increased ADA activity. Treatment with the ADA inhibitor deoxycoformycin (DCF) is able to reverse the effects of MPTP (Huang et al., 2019). However, in lysosomes of ADA mutant B-lymphocytes, adenosine accumulation impairs TRPML1 activity and triggers lysosome enlargement and dysfunction (Zhong et al., 2017). These data suggest that the lack of TRPML1 activity could lead to an increased susceptibility to oxidative stress and cell death. Therefore, rigorous experiments should be conducted to further explore the possible role of TRPML1 as a therapeutic target in PD.

## Amyotrophic Lateral Sclerosis

**FIGURE 1:**
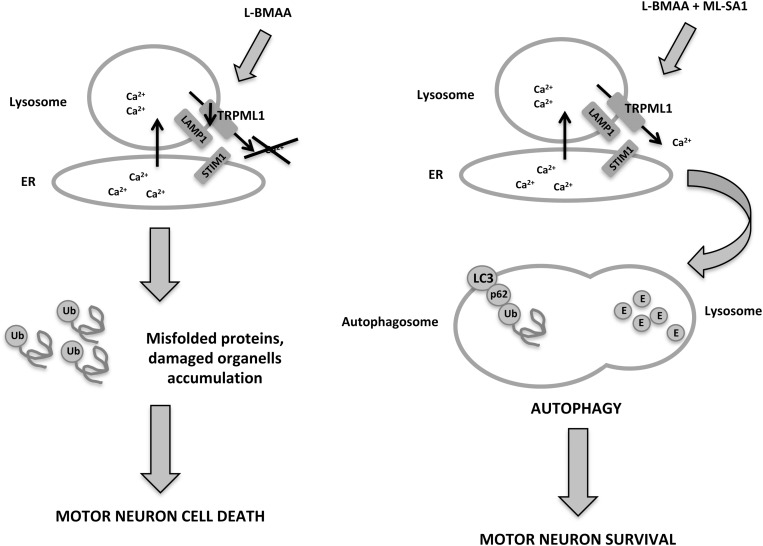
In a neurotoxin L-BMAA-induced ALS mouse model, TRPML1 is downregulated, autophagy is impaired and motor neurons die from accumulation of misfolded proteins. However, administration of the TRPML1 agonist, ML-SA1, activates the channel and leads to lysosomal Ca^2+^ release, autophagic flux and motor neuron survival.

Amyotrophic lateral sclerosis (ALS) is a neurodegenerative disease that leads to progressive loss of motor neurons in the anterior horn of the spinal cord, with muscle weakness, wasting, and spasticity (Kiernan et al., 2011). It is classified as either sporadic or familial: for familial ALS mutations in superoxide dismutase 1 (SOD1) enzyme, TAR DNA binding protein 43 and proteins involved in autophagic pathway and lysosome function are present (Chen et al., 2013). The latter two are regulated by intracellular Ca^2+^ flux inside the cell. In particular, lysosomal Ca^2+^ can be released by intracellular signals, such as NAADP (Kauppila et al., 2017) and PI(3,5)P2 (López-Otín et al., 2013). TRPML1 could play an important role in restoring autophagy and lysosome function in ALS, given that Ca^2+^ release is crucial for lysosome function, that TRPML1 is implicated in lysosomal Ca^2+^ release, and that PI(3,5)P2 levels are impaired in ALS. In the ALS mouse model, chronic exposure to the neurotoxin L-BMAA impairs autophagy in primary motor neurons, leading to ER stress and cell death (Tedeschi et al., 2019a; Figure 1). In these neurons, TRPML1 protein levels are downregulated; however, early channel activation induced by the ML-SA1 agonist is able to counteract TRPML1 impairment and reduce ER stress proteins and Caspase-9 upregulation, thus rescuing motor neurons from death. Under normal conditions in motor neuronal cells, the lysosomal TRPML1 colocalizes with the ER Ca^2+^ sensor STIM1, which suggests that there is cross-talk between ER and lysosomes, in which lysosomal Ca^2+^ efflux through TRPML1 plays a pivotal role (Tedeschi et al., 2019b). The depletion of ER Ca^2+^ stores affects the lysosomal Ca^2+^ release that takes place through the action of TRPML1. These data suggest that ER is a key source of lysosomal Ca^2+^ in motor neurons, as demonstrated also in HEK 293 cells that stably express GCaMP3-TRPML1 (Garrity et al., 2016); altered Ca^2+^ homeostasis in one of these organelles has dramatic implications on the other stores (Tedeschi et al., 2019a).

Furthermore, ER dysfunction is common to different forms of ALS, from sporadic ALS, which is characterized by misfolding of wild-type SOD1, to the L-BMAA-induced ALS mouse model. Tedeschi et al. demonstrated that the agonist ML-SA1 is able to prevent the increase of ER stress markers. Thus it can be assumed that the proximity to ER store and lysosomes means that lysosomal Ca^2+^ release through TRPML1 may contribute to ER Ca^2+^ concentration and stress prevention by continuous refilling of Ca^2+^ (Tedeschi et al., 2019b).

## Mucolipidosis Type IV

Mucolipidosis type IV (MLIV) is an autosomal recessive lysosomal storage disorder due to *MCOLN1* gene mutations (Bargal et al., 2000; Sun et al., 2000). Neurodegeneration with spasticity, hypotonia, and the inability to walk independently are common hallmarks (Altarescu et al., 2002).

Some publications have connected TRPML1 mutations with the lower lysosomal pH registered in MLIV patients compared with normal control (Raychowdhury et al., 2004; Soyombo et al., 2006), although these results are different from data reported by Bach (Bach et al., 1999). In particular, Soyombo et al. has demonstrated that TRPML1 is able to prevent lysosomal overacidification because it is permeable to H^+^ and thus it dissipates high H^+^ concentration to maintain lysosomal homeostasis under normal condition (Soyombo et al., 2006). In the absence of TRPML1 regulation of pH, the acidic conditions result in the dysfunction of lysosomal hydrolase and thus substrates accumulation.

Given its permeability to Ca^2+^, TRPML1 activation is required to allow the attachment of vesicles to motor proteins along the microtubules and the fusion with plasma membrane in normal cells. In MLIV, the loss of TRPML1 function is related to defects in lysosomal biogenesis and exocitosis (LaPlante et al., 2006).

Fibroblasts of MLIV patients contain soluble protein and lipid aggregates (Bach, 2001; Altarescu et al., 2002; Smith et al., 2002) due to abnormal sorting and/or transport of these macromolecules along the late endocytic pathway (Bargal and Bach, 1997; Chen et al., 1998). Typical aspects of MLIV are mitochondrial fragmentation and decreased mitochondrial Ca^2+^ buffering efficiency (Jennings et al., 2006; Kiselyov et al., 2007b). Since lysosomes are significant players in the autophagic recycling of mitochondria, defects in their function may affect recycling and thus lead to the storage of fragmented mitochondria and the failure to buffer cytoplasmic Ca^2+^. The reduced buffering capacity could make cells more sensitive to pro-apoptotic signals (Kiselyov et al., 2007a; Venugopal et al., 2009; Demers-Lamarche et al., 2016).

As described above, TRPML1 induces TFEB transcriptional activity, and TRPML1 is itself the target of TFEB (Medina et al., 2015). This creates a feedback loop that activates autophagy. In addition, a new TFEB-independent pathway has been demonstrated (Scotto Rosato et al., 2019). Acute activation of TRPML1 is able to increase phagophore formation, thus activating calcium/calmodulin-dependent protein kinase kinase β (CaMKKβ) and AMP-activated protein kinase (AMPK), and also inducing the formation of the Beclin1/VPS34 autophagic complex and the production of phosphatidylinositol 3-phosphate (PI3P). PI3P-enriched ER subdomains act as platforms for phagophore formation. These results are of considerable importance because in the cells of MLIV patients, defective production of PIP3 impairs recruitment of PI3P-binding proteins (WIPI2 and DFCP1) to the phagophore during autophagy induction.

Moreover, TRPML1 could also have a role in the preservation membrane potential useful for the efficient transport of chaperone-mediated autophagy (CMA) substrate proteins for degradation. The intraluminal loop of TRPML1 seems to interact directly with heat shock cognate protein of 70 kDa and heat shock proteins of 40 kDa, members of a molecular chaperone complex required for protein transport into the lysosome during CMA (Venugopal et al., 2009). Of note, lysosomes from MLIV patients exhibit a reduction in CMA. Also, in MLIV lysosomes there is a reduced amount of lysosomal-associated membrane protein type 2A essential for the chaperones complex bound to the lysosome membrane. Related to the impairment of CMA, MLIV fibroblasts increase the oxidized protein levels that sensitize neurons to apoptosis, thus leading to neuronal degeneration (Venugopal et al., 2009).

Neurodegenerative effects have been also correlated to zinc accumulation in lysosomes in MLIV fibroblasts or in *TRPML1*-knockdown HEK-293 cells (Eichelsdoerfer et al., 2010). This accumulation is not reverted by treatment with the TRPML1 agonist MK6-83; in contrast, treatment with MK6-83 significantly reduces zinc accumulation in F408del TRPML1 mutant-expressing fibroblasts (Chen et al., 2014).

Several patients show *MCOLN1* gene mutations that introduce premature stop signals and result in an absent TRPML1 protein, or a protein lacking the ion-conducting pore between TMD5 and TMD6. Some have single point mutations that maintain an open reading frame (Altarescu et al., 2002; Manzoni et al., 2004), some have mislocalized proteins, some have TRPML1 correctly localized but incapable of responding to endogenous ligands. In the latter situation, there may be promise in therapy based on the use of an agonist of TRPML1 to enhance its activity. Indeed, *in vitro* results demonstrated that small-molecule ligands are able to recover endogenous channel activity and also endo-lysosomal trafficking defects and accumulation of zinc (Chen et al., 2014).

## Niemann-Pick Disease

Niemann-Pick diseases (NPD) are lipid storage pathologies associated with central nervous system impairment due to lipid accumulation (Patterson and Walkley, 2017; Torres et al., 2017). Three types of NPD have been identified. Types A and B are characterized by deficient activity of acid sphingomyelinase, which degrades lysosomal sphingomyelin; type C shows defective function in cholesterol efflux from lysosomes (Patterson and Walkley, 2017; Schuchman and Desnick, 2017; Torres et al., 2017) as a consequence of mutation in *NPD type C1 (NPC1)* or *NPD type C2 (NPC2)* genes, responsible for cholesterol transport. This causes an increase in concentration of cholesterol with accumulation of unesterified cholesterol in late endosomes/lysosomes. Accumulation of sphingomyelin and cholesterol affects lysosomal Ca^2+^ release and blocks endocytosis and fusion between late endosomes and lysosomes, resulting in endocytosis and autophagy dysfunction (Samie and Xu, 2014; Höglinger et al., 2019; Tancini et al., 2019).

Sphingomyelin is able to inhibit Ca^2+^ efflux through the TRPML1 channel. Therefore, by inhibiting TRPML1 activity, the accumulation of sphingomyelin could influence both lysosomal pH and Ca^2+^ signaling through ER and mitochondria (Lloyd-Evans and Platt, 2010; Wheeler et al., 2019). Moreover, TRPML1 forms a complex with the large conductance Ca^2+^-activated K^+^ channels (BK) in lysosomes. The BK channels are activated by TRPML1-mediated Ca^2+^ release to maintain the negative membrane potential needed for sustained lysosomal Ca^2+^ release (Cao et al., 2015). Either TRPML1 or BK deficiency results in lysosomal Ca^2+^ accumulation, defective lysosomal membrane trafficking, and lysosome storage. Furthermore, upregulation of TRPML1 or BK reverses the impaired lysosome Ca^2+^ release and membrane trafficking in NPC1 fibroblasts. Moreover, in NPC1 or NPC2 KO HeLa cells, cholesterol accumulates in late endosomes, and the treatment with 2-hydroxypropyl-ß-cyclodextrin reduces cholesterol content (Vacca et al., 2019). Here TRPML1 silencing abrogates this effect: this may suggest that TRPML1 is directly implicated in the regulation of endo-lysosome secretion.

## Discussion

Neurodegenerative diseases are serious health problems. Numerous efforts have been made to identify neuropathological, biochemical and genetic biomarkers for them. Mitochondrial function and resistance to oxidative stress are compromised during the aging phase, and this is a starting point for the onset of neurodegenerative diseases (Cenini et al., 2019). Other factors that promote oxidative stress are excitotoxicity and aberrant protein processing, which lead to outcomes such as impairment of lysosome integrity. Given that lysosomes are the major contributors to autophagic recycling of mitochondria, to misfolded protein and to damaged organelles, it may be that defects in lysosome function affect mitochondrial recycling, cause accumulation of fragmented mitochondria, and block the ability to buffer cytoplasmic Ca^2+^, and that these processes in turn sensitize cells to pro-apoptotic signals. In this regard, several reports suggest that lysosomal Ca^2+^ impairment is involved in the pathogenesis of neurodegenerative diseases. For this reason, the calcium channels expressed on lysosomes have been attracting a lot of attention lately, especially as potential new targets for fighting neurodegeneration. It is now well known that TPCs and TRPMLs are the two main calcium permeable receptor families expressed on lysosomes. However, the pharmacology of these receptors has not yet been well elucidated and still requires further studies. In addition, new findings are necessary to clarify if the Ca^2+^ efflux from lysosomes is helpful or not. In fact, contradictory data are present in the scientific literature. According to some researchers, the accumulation of calcium in lysosomes seems to be associated with lysosomal dysfunctions in neurodegenerative diseases (Bae et al., 2014). Others, instead, suggest that the inhibition of the NAADP-induced Ca^2+^ mobilization is beneficial in some experimental models of neurodegenerative diseases. It is well established that the NAADP-induced lysosomal Ca^2+^ efflux is dependent on TPCs (Brailoiu et al., 2010; Yamaguchi et al., 2011; Morgan et al., 2015; Pitt et al., 2016; Grimm et al., 2017; Patel et al., 2017). However, recent findings also indicate that TRPML1 is a target of NAADP, thus supporting the view that it plays a role in endosome/lysosome interaction, lipid trafficking and alterations in autophagy machinery (Lee et al., 2015). In fact, it has also been shown that in a *MCOLN1*^–/–^ fibroblast model, NAADP action is abolished, an observation that suggests that NAADP-TRPML1 signaling plays a significant role (Zhang et al., 2011).

**FIGURE 2:**
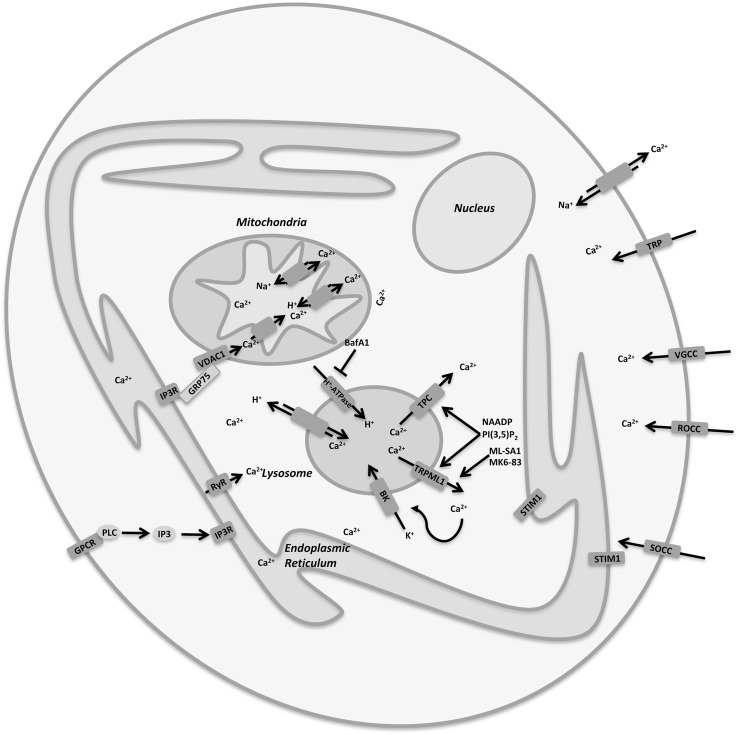
Cellular Ca^2+^ homeostasis is regulated by a complex interplay between plasma membrane and organelles. Lysosomes are important organelles directly involved in Ca^2+^ signaling and homeostasis and express a variety of Ca^2+^ channels, including TRPML1 and TPCs.

In neurons, the regulation of Ca^2+^ concentrations in each cellular compartment is essential for the maintenance of normal cellular functions and for neuronal plasticity (Ureshino et al., 2019). Ca^2+^ buffering is controlled by the interplay between ER, mitochondria and lysosomes that express Ca^2+^ transport mechanisms such as TRPML channels. Moreover, Ca^2+^ mobilization is regulated by several cation channels expressed in the plasma membrane involved in the cation exchange with the microenvironment. It is definitively clear that the imbalance of Ca^2+^ concentrations is strongly involved in the pathogenesis of neurodegenerative diseases, as in these pathologies there is often an evident defect in intracellular calcium storage. In fact, in many different experimental models of neurodegeneration, Ca^2+^ mobilization from organelles to cytoplasm or vice versa is impaired. However, it is still difficult to clarify whether Ca^2+^ plays the same role in the different neuronal disorders, especially because it functions as a messenger in an intricate network regulated by the ER/mitochondria/lysosome axis involving both pro-survival and death pathways (Figure 2; Ureshino et al., 2019). Therefore, calcium dyshomeostasis in both lysosome and cytoplasm is detrimental. In this regard, there is no doubt that channel dysfunctions are manifest in vesicular trafficking defects, and further work is required to delineate the affected processes more precisely.

As shown in this review, calcium imbalance, lysosomes and oxidative stress, as well as the function of TRPML1 seem to be highly significant in the neurodegenerative diseases described. Unfortunately, to date little data is available linking TRPML channels and neurodegeneration, and more studies are needed in order to clarify the role of these channels. In conclusion, a deeper understanding of the exact mechanism of neurodegeneration will offer a valid starting point for the development of new therapeutic strategies, and in this regard TRPML1 is turning out to be a key candidate.

## Author Contributions

GS and MM contributed to the conception and design. MM, FM, and CA drafted the manuscript. OM, MN, and GS contributed to the critical revision of the manuscript. MM and CA re-examined and revised the manuscript.

## Conflict of Interest

The authors declare that the research was conducted in the absence of any commercial or financial relationships that could be construed as a potential conflict of interest.
